# Chromosomal Numerical Aberrations and Rare Copy Number Variation in Patients with Inflammatory Bowel Disease

**DOI:** 10.1093/ecco-jcc/jjac103

**Published:** 2022-07-30

**Authors:** Paulina Dirvanskyte, Bhaskar Gurram, Chrissy Bolton, Neil Warner, Kelsey D J Jones, Helen R Griffin, Genomics England Research Consortium, Jason Y Park, Klaus-Michael Keller, Kimberly C Gilmour, Sophie Hambleton, Aleixo M Muise, Christian Wysocki, Holm H Uhlig

**Affiliations:** Translational Gastroenterology Unit and Biomedical Research Centre, Nuffield Department of Clinical Medicine, University of Oxford, Oxford, UK; Department of Pediatrics, UT Southwestern Medical Center, Dallas TX, USA; Institute of Child Health, University College London, London, UK; Paediatric Gastroenterology Department, Great Ormond Street Hospital for Children NHS Foundation Trust, London, UK; SickKids Inflammatory Bowel Disease Centre, Hospital for Sick Children, Toronto, ON, Canada; Paediatric Gastroenterology Department, Great Ormond Street Hospital for Children NHS Foundation Trust, London, UK; Nuffield Department of Orthopaedics, Rheumatology and Musculoskeletal Sciences, Kennedy Institute of Rheumatology, University of Oxford, Oxford, UK; Primary Immunodeficiency Group, Newcastle University Translational and Clinical Research Institute, Newcastle upon Tyne, UK; Genomics England, London, UK; Department of Pathology and the Eugene McDermott Center for Human Growth and Development. UT Southwestern Medical Center, Dallas, TX, USA; German Clinic for Diagnostics [DKD], Helios Klinik, Wiesbaden, Germany; Laboratory of Immunology and Cellular Therapy, Great Ormond Street Hospital for Children, NHS Foundation Trust, London, UK; Primary Immunodeficiency Group, Newcastle University Translational and Clinical Research Institute, Newcastle upon Tyne, UK; SickKids Inflammatory Bowel Disease Centre, Hospital for Sick Children, Toronto, ON, Canada; Department of Biochemistry, University of Toronto, Toronto, ON, Canada; Cell Biology Program, Sick Kids Research Institute, Hospital for Sick Children, Toronto, ON, Canada; Department of Pediatrics, University of Toronto, Toronto, ON, Canada; Institute of Medical Science, University of Toronto, Toronto, ON, Canada; Department of Pediatrics, and Department of Internal Medicine, University of Texas Southwestern Medical Center, Dallas, TX, USA; Translational Gastroenterology Unit and Biomedical Research Centre, Nuffield Department of Clinical Medicine, University of Oxford, Oxford, UK; Biomedical Research Centre, University of Oxford, Oxford, UK; Department of Paediatrics, John Radcliffe Hospital, Oxford, UK

## Abstract

**Background and Aims:**

Inflammatory bowel diseases [IBD] have a complex polygenic aetiology. Rare genetic variants can cause monogenic intestinal inflammation. The impact of chromosomal aberrations and large structural abnormalities on IBD susceptibility is not clear. We aimed to comprehensively characterise the phenotype and prevalence of patients with IBD who possess rare numerical and structural chromosomal abnormalities.

**Methods:**

We performed a systematic literature search of databases PubMed and Embase; and analysed gnomAD, Clinvar, the 100 000 Genomes Project, and DECIPHER databases. Further, we analysed international paediatric IBD cohorts to investigate the role of *IL2RA* duplications in IBD susceptibility.

**Results:**

A meta-analysis suggests that monosomy X [Turner syndrome] is associated with increased expressivity of IBD that exceeds the population baseline (1.86%, 95% confidence interval [CI] 1.48 to 2.34%) and causes a younger age of IBD onset. There is little evidence that Klinefelter syndrome, Trisomy 21, Trisomy 18, mosaic Trisomy 9 and 16, or partial trisomies contribute to IBD susceptibility. Copy number analysis studies suggest inconsistent results. Monoallelic loss of X-linked or haploinsufficient genes is associated with IBD by hemizygous or heterozygous deletions, respectively. However, haploinsufficient gene deletions are detected in healthy reference populations, suggesting that the expressivity of IBD might be overestimated. One duplication that has previously been identified as potentially contributing to IBD risk involves the *IL2RA/IL15R* loci. Here we provide additional evidence that a microduplication of this locus may predispose to very-early-onset IBD by identifying a second case in a distinct kindred. However, the penetrance of intestinal inflammation in this genetic aberration is low [<2.6%].

**Conclusions:**

Turner syndrome is associated with increased susceptibility to intestinal inflammation. Duplication of the *IL2RA/IL15R* loci may contribute to disease risk.

## Graphical Abstract

**Figure:**
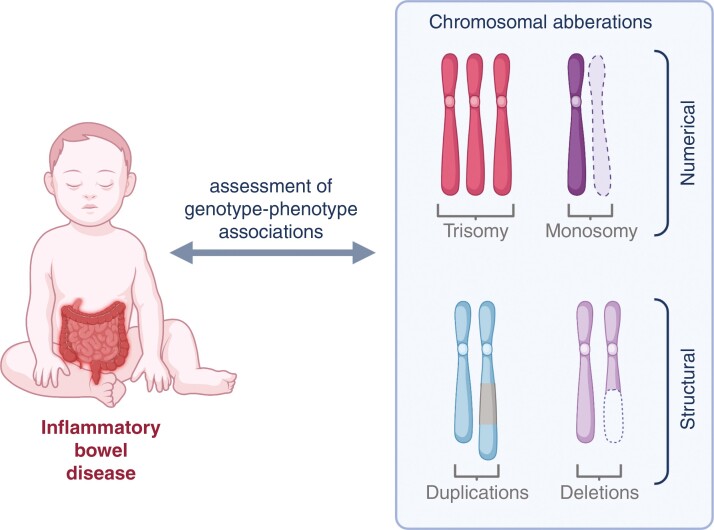

## 1. Introduction

Inflammatory bowel disease [IBD] comprises a group of inflammatory disorders arising from the dysregulated interplay between innate and adaptive immune responses and the gut environment, including intestinal microbiota and dietary factors.^1,2^ IBD is categorised by endoscopic and histological features into Crohn’s disease [CD], ulcerative colitis [UC], and IBD unclassified [IBDU]. Genome-wide association studies have uncovered hundreds of common polygenic risk loci that contribute towards the genetic heritability of classical polygenic IBD.^3^ However, rare genetic variants may also contribute significantly to the risk of an individual developing classical polygenic IBD.^4^ In contrast to the majority of patients with polygenic IBD, IBD may also emerge with a Mendelian pattern of inheritance, where a single gene defect leads to IBD.^5^ In a recent position statement of the European Society for Paediatric Gastroenterology Hepatology and Nutrition [ESPGHAN], 75 genes causing monogenic IBD were highlighted as having clinical significance for diagnosis and treatment.^6^ Overall, over 100 genes likely contribute to monogenic forms of IBD.^7^ These genes encode a functionally diverse set of proteins, and defects may cause primary immunodeficiency, immune dysregulation, and intestinal epithelial dysfunction, among other phenotypes. Several monogenic IBD syndromes are associated with particularly severe and treatment-refractory disease that can be associated with significant morbidity.^6^

There is limited knowledge regarding whether larger structural and numerical chromosomal abnormalities affect IBD susceptibility. The structural diversity of the human genome encompasses complete or partial loss or gain of entire chromosomes, such as monosomies and trisomies, and includes smaller deletions or duplications that contribute to copy number variation [CNV]. CNV contributes towards 4.8–9.7% of the diversity of the human genome,^8^ suggesting a relevant contribution to disease, especially for dosage-sensitive genes.^9^ Despite this, the contribution of CNV in monogenic IBD genes has been scarcely investigated in genetic susceptibility studies.

Here, we perform a systematic search on the role of numerical chromosomal abnormalities and rare CNV. We review the literature and interrogate the DECIPHER and ClinVar databases to identify case reports of genetic structural aberrations linked to IBD. We identify candidate regions for rare CNV and perform a replication analysis in large IBD cohorts, based on exome sequencing.

## 2. Methods

### 2.1. Ethics approval

Experiments were carried out with Research Ethics Board [REB] approval from the Hospital for Sick Children, the Oxford IBD cohort study [rare disease subproject], the Department of Pediatrics, UT Southwestern Medical Center, and Genomics England Research Consortium. Informed written consent to participate in research was obtained from patient/families and controls. Parents of the patients that are reported as case report in this paper consented to their information being published as a detailed case report.

### 2.2. Definitions

Genomic chromosomal abnormalities were categorised into numerical chromosomal abnormalities and structural variants. Numerical abnormalities were defined as chromosomal aneuploidies where the entire chromosome was either gained or lost. These were further divided into sex chromosome aneuploidies [monosomy X, ie, Turner syndrome; and 47,XXY, ie, Klinefelter syndrome] and trisomies [Trisomy 21, Trisomy 18, Trisomy 16, and Trisomy 9]. Structural abnormalities were defined as unbalanced structural rearrangements that result in CNV larger than 1 kb in size.^10^ These were further divided into partial monosomies and trisomies that were considered to be: >1 Mb [large deletions or duplications] or <1 Mb [microdeletions and microduplications]. Rare CNVs were defined as present at mean allele frequencies <0.001 based on the data from the gnomAD database.^11^ More common CNVs <1 kb in size were excluded from the search, which was designed to focus on rare and putatively highly penetrant variants and to focus on gene dose effects.

### 2.3. Search strategy

**Figure 1.**
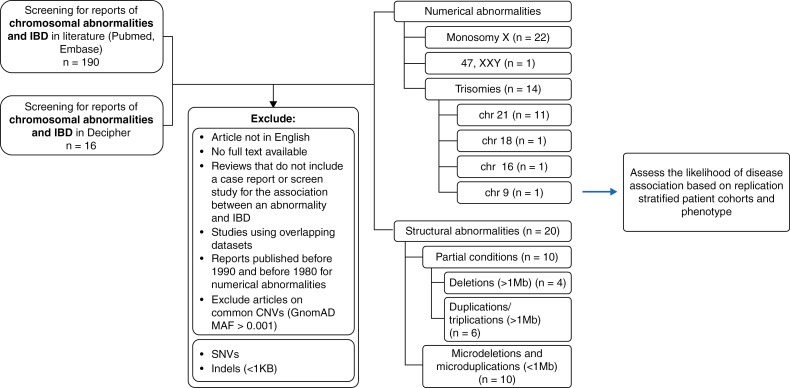
Reports identified and excluded in systematic review of cytogenetic abnormalities and inflammatory bowel disease [IBD]. Six disorders or syndromes were included in the initial assessment. Reports of IBD or IBD-like disease were the initial selection criteria that yielded 190 publications and 16 DECIPHER entries. Reports were excluded based on exclusion criteria. Numbers in brackets represent the number of cases for each abnormality type that remained after exclusion. These numbers include duplicated publications or Decipher reports that include more than one abnormality. The total number of unique publications and Decipher reports was 50.

We performed a systematic literature search on the role of numerical and structural chromosomal abnormalities in patients with IBD. PubMed and Embase databases were searched for each of the chromosomal defects and CNVs separately [last accessed August 31, 2021] [Figure 1]. The search was done for terms ‘inflammatory bowel disease’ and ‘Turner syndrome’, ‘Down syndrome’, ‘Klinefelter syndrome’, ‘trisomy 9’, ‘trisomy 18’, ‘trisomy 16’, ‘partial trisomy’, ‘partial monosomy’, ‘copy number variation’. These were mapped to Medical Subject Heading controlled vocabulary terms on PubMed or mapped to Emtree terms on Embase [Supplementary Figure 1]. The search was limited to publication years 1980–2021 for chromosomal aneuploidies and 1990–2021 for structural abnormalities, to acknowledge technical progress in detection of CNV using cytogenetics and hybridisation technologies.

Abstracts were screened to remove irrelevant results that did not include the search terms. Only full-text papers written in English were included. Additional studies were identified from conferences, correspondence with authors, and manual searching of included study references and citations.

### 2.4. DECIPHER database search

The literature search was complemented by analysis of genetic aberrations associated with colitis, enterocolitis, UC, and CD in the DECIPHER and ClinVar databases [Figure 1]. The DECIPHER database [release DECIPHER v11.6 on August 18, 2021, https://www.deciphergenomics.org]^12^ contains data from over 39 000 patients who provided consent for broad data sharing. Single nucleotide variants and insertions/deletions less than 1 kb in length were excluded from the search. For CNV analysis of specific regions, additional patient data were analysed from the ClinVar database, which contains reports on human genetic variation and its relationship to clinically relevant phenotypes [accessed on August 18, 2021, https://www.ncbi.nlm.nih.gov/clinvar/]. ^13^

To assess the frequency of genetic variants in a healthy population, we screened the GnomAD database [release v3.1.3 September 2021, https://gnomad.broadinstitute.org/]. GnomAD removes data from patients with severe paediatric disease and their first-degree relatives, so their frequency in the database is lower than in the general population.^11^

### 2.5. Replication study to detect patients with IL2RA duplication

A survey for patients with *IL2RA* duplication was performed across several IBD cohorts. Patients with *IL2RA* duplications were screened for in tertiary referral centre databases in Oxford, UK [*n* = 700 genotyped IBD patients], London UK [*n* = 1296],^14^ Toronto, Canada [*n* = 3158],^15^ and in the UK 100 000 Genomes Project database which contains whole-genome data from individuals with rare disease or cancer.^16^ In addition, we have reached out to key centres of the very-early-onset IBD consortium [https://veoibd.org] to see whether additional patients have been clinically identified [focusing on paediatric gastroenterology centres].

For a formal search of patients with *IL2RA* duplications in the Toronto IBD cohort, we screened exome sequencing data from two cohorts of samples totalling 3158 individuals [1293 patients with paediatric IBD] and their first-degree relatives [parents and siblings when available]. The samples were sequenced by the Regeneron Genetic Center and analysed for CNVs using CLAMMs^17^ [https://github.com/rgcgithub/clamms]. Outlier samples were removed, leaving on average 11 high-confidence CNV calls per sample. The majority [80%] of the samples used in the CNV analysis come from a previously published cohort collected from the greater Toronto area.^15^

The Genomics England 100 000 genomes dataset was also screened for duplications that included *IL2RA*. Copy number gains were called using Canvas.^18^ The structural variant calls of rare disease probands and relatives [64 058 genomes] and cancer participant germline sequences [15 677 genomes] were aligned to the GRCh38 [Genome Reference Consortium Human Build 38] reference genome and filtered for ‘PASS’ quality duplications with quality score >10 and length >10 Kb.

### 2.6. *IL2RA* expression analysis, lymphocyte subsets, and proliferation assays

Flow cytometric analysis of lymphocyte subsets, maturation, and activation markers was performed using BD Canto and Canto II flow cytometers with FACSDiva software. Cell staining protocols included BD Multitest 4-Color and 6-Color TBNK Reagent with BD Trucount Tubes with additional cell surface markers and fluorochrome conjugates added [from BD]: TCR αβ FITC [clone WT31], TCR γδ PE [clone 11F2], HLA-DR PerCP Cy5.5 [clone L243], HLA-ABC FITC [clone G46-2.6], Beta-2 microglobulin PE [clone TU99], CD45RA FITC [clone L48], CD2 PE [clone S5.2], CD45RO APC [clone UCHL-1], CD45RA FITC [clone L48], CD40 APC [Life Technologies, clone HB14], and CD62L PE [Life Technologies, clone DREG56]. The percentage of CD4 + HLA-DR + T cells was calculated as percentage of total HLA-DR + lymphocytes minus percentage of total CD19 + lymphocytes. For analysis of regulatory and effector T cell subsets, cells were permeabilised and stained with anti-FoxP3 FITC [Ebioscences, clone PCH101]. Surface marker stains used CD4 PerCP [BD, clone SK3], CD25 APC [BD, clone 2A3], and CD127 PE [Immunotech/Beckman Coulter, clone R34.34]. All markers were evaluated against isotypic controls.

STAT5 phosphorylation in response to IL2 in stimulated T cells was assessed by flow cytometry with a BD FACSLyric flow cytometer and FCS Express software. Whole blood was incubated with IL2 or no IL2 for 10 min. Cells were permeabilised and stained with anti-CD4 PerCP [BD clone SK3] and anti-pSTAT5 [pY694] [Alexa Fluor 488, BD clone 47].

Lymphocyte proliferation was quantified by flow cytometry of fluorescently dyed alkyne-modified nucleoside incorporation in peripheral blood mononuclear cells [PBMCs] that were stimulated with three different stimuli: agonistic anti-CD3 alone, agonistic anti-CD3 plus anti-CD28, and agonistic anti-CD3 plus exogenous IL2. Proliferation was measured as the amount of fluorescent signal.

A control sample from a healthy unrelated individual was included for all analyses.

### 2.7. Statistics

The 95% confidence intervals [CIs] for each proportion were calculated using the Wald method. The Mann–Whitney test was used for comparing age of onset data between the classical IBD and Turner syndrome cohorts. One-way analysis of variance [ANOVA] was used to compare age of onset date between different sub-genotypes of the Turner syndrome cohort. All statistical analyses were done using the GraphPad PRISM software [version 9.2.0].

## 3. Results

### 3.1. Numerical chromosomal aberrations

**Figure 2.**
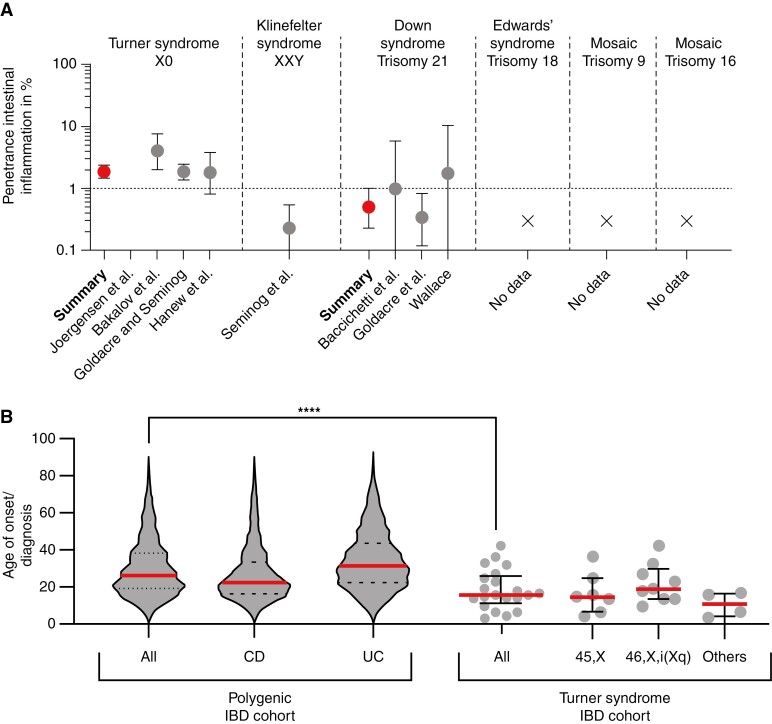
Phenotype of individuals with large chromosomal aberrations and inflammatory bowel disease [IBD]. [A] Penetrance of intestinal inflammation. Penetrance [dot] and 95% confidence intervals [bars] were calculated using the modified Wald equation. [B] Age of onset of intestinal inflammation in the polygenic IBD and Turner syndrome cohorts. Left: polygenic IBD cohort [total *n* = 1762]. Right: Turner syndrome cohort by sub-genotype. Each dot represents one patient [total *n* = 21]. ****p <0.0001, Mann–Whitney U test. Lines represent the medians and bars/dotted lines represent interquartile range.

The database search identified 41 unique relevant articles that were reviewed in full text and nine unique DECIPHER reports [Figure 1]. Two unique articles reported on multiple conditions^19,20^ as well as one Decipher entry [Patient 349797], so they were included as duplicates. In patients with Turner syndrome the reported penetrance exceeded the conservative upper border population baseline risk for IBD of 1%.^21^ There was insufficient evidence to support the association between IBD and Down syndrome, Klinefelter syndrome, Trisomy 9, Trisomy 18, Trisomy 16, or large chromosomal deletions or duplications [Figure 2A].

### 3.2. Turner syndrome is associated with a penetrance of IBD that exceeds the population baseline

In total 22 published studies were identified describing patients with Turner syndrome and IBD [Figure 1]; 23 individuals with IBD and Turner syndrome were described in case reports [Supplementary Table 1]. Four larger cohort studies summarised the phenotypes of 3866 patients with Turner syndrome, and 72 of these individuals exhibited IBD, allowing the estimation of penetrance across unselected cohorts^22–25^ [Supplementary Table 1]. Individuals with Turner syndrome had a moderate penetrance of IBD of 1.86% [95% CI 1.48 to 2.34, Figure 2A]. The mean age of diagnosis or onset of IBD in the Turner syndrome cohort was 17.8 ± 2.3 standard error of the mean [SEM] [range: from 3 to 41 years], which was significantly younger than the IBD onset age in the Oxford IBD cohort^5,7^ [*p* <0.0001, Mann–Whitney test] [Figure 2B].

Data from the Turner syndrome case reports and Turner syndrome cohort studies did not show an enrichment of a particular IBD phenotype. CD was reported in 65% of case reports, and similarly the larger-scale studies did not suggest a strong bias towards either phenotype.^23–25^ Based on the case reports, 26% of patients with Turner syndrome [6/23] were described to have a ‘severe’ phenotype of IBD. However, this may be a consequence of publication bias. Consistent criteria for assessing severity were lacking and an increased severity was not reported in the Turner syndrome cohort studies. We investigated whether sub-genotypes of patients with Turner syndrome carried a different risk profile. Among the case reports [*n* = 22], 27% of individuals were 45,X; 18% were 46,X,i[Xq]; 23% expressed 45,X/46,X,i[Xq] mosaicism; and 32% had other forms of mosaicism. We did not find a significant correlation between karyotype and IBD onset age [*n* = 20, *p* = 0.1859, ANOVA] [Figure 2B]. Among the Turner syndrome cohort studies,^22,25^ 9/18 [50%] of Turner syndrome patients with IBD had an i[Xq] genotype [including both mosaic and complete genotypes]. The odds ratio for association of IBD with the 46,X,i[Xq] genotype was 2.11 [95% CI 1.15 to 3.88] compared with a population of those with Turner syndrome and no IBD.^26^

### 3.3. Klinefelter syndrome

One paper reported five patients with Klinefelter syndrome [47, XXY] and UC, out of a cohort of 2208 patients.^27^ The relative risk [RR] was not significantly elevated for this condition [RR = 1.1, 95% CI 0.3 to 2.4].

### 3.4. Down syndrome and IBD

In all, 21 patients with IBD and trisomy 21 [Down syndrome] were reported [Supplementary Table 1]. Two isolated reports suggest a combination of primary sclerosing cholangitis and Crohn’s disease in patients with Down Syndrome.^28,29^ In a large IBD cohort, the prevalence of Down syndrome was similar to that in the general population [of Spain 0.2% vs 0.1%, respectively, *n* = 1200].^30^ Based on all the published data of individuals with Down syndrome and IBD, we found no association with a particular IBD sub-phenotype [Figure 2A]. Age of IBD onset was 25 years +/- 5.4 for males and 12 years +/- 2.3 for females [based on *n* = 4 and *n* = 8, respectively]. There was insufficient evidence for a younger age of onset or increased severity in patients with Trisomy 21. In summary, there was no evidence for increased IBD susceptibility in this population.

### 3.5. Trisomy 9, Trisomy 18, and Trisomy 16

Three patients with either full Trisomy 18, mosaic Trisomy 9, or Trisomy 16 were identified who presented with IBD [Supplementary Table 1]. We identified a 2-year-old child with Trisomy 18, characterised by discontinuous colitis with histopathological changes of chronic active inflammation with mild architectural distortion consistent with IBDU [Supplementary Information]. A child with Trisomy 9 was described to suffer from CD from the age of 2 years, and the patient with mosaic Trisomy 16 had UC onset at 10 years of age.^31,32^ A strong association between IBD and these syndromes cannot be assumed, given the large number of patients reported with these genetic conditions without intestinal inflammation.^33–35^

### 3.6. Deletions that impact on monogenic IBD genes

Several case reports identified *de novo* deletions involving known gene loci for monogenic IBD [Supplementary Table 2].^36–39^ Structural abnormalities suggesting a loss of gene function were identified for genes where hemizygous or heterozygous variants have been implicated in causing monogenic IBD. Exemplars included deletions of the *CTLA-4* locus,^36^*TNFAIP3* locus,^37^ and hemizygous deletions of *XIAP*.^38^ Another case report described three patients with IBD and homozygous loss of function of the *IL10RB* locus: two patients with homozygous deletions and a patient with a heterozygous deletion and a duplication causing a frameshift loss-of-function mutation.^39^ Recently, a genome sequencing identified a 12.3 kb homozygous tandem duplication that disrupted the reading frame of the *LRBA* gene.^40^

In DECIPHER, 20 patients had a *CTLA4* deletion, 10 had a *TNFAIP3* deletion, and 30 had X-linked *XIAP* deletions, including one 46,XY patient. Out of these patients, none presented with IBD-like disease and two patients with *CTLA4* deletion and one patient with *TNFAIP3* deletion had ‘recurrent infections’ listed as a phenotype. The 46,XY patient with the *XIAP* deletion had mild global developmental delay but no symptoms characteristic of X-linked lymphoproliferative syndrome 2 [XLP2] caused by *XIAP* deficiency. The lack of IBD or other immunodeficiency symptoms in patients with these deletions confirms the incomplete expressivity or the patients are listed on the database before immunodeficiency manifests.

### 3.7. CNV and IBD

**Figure 3.**
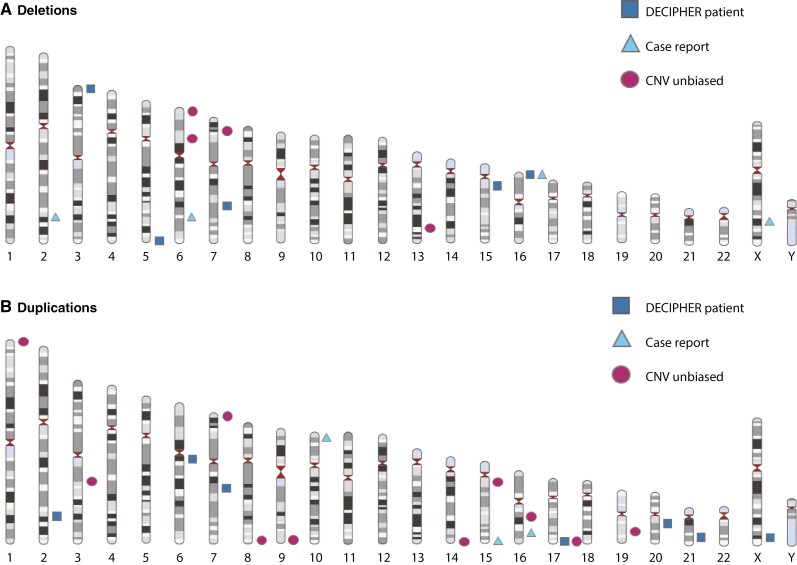
Structural abnormalities and intestinal inflammation. [A] Deletions. Overview of different studies and case reports. [B] Duplications. Overview of different studies and case reports.

Two studies investigated the association between rare genome-wide CNV [population frequency <0.1%^41^ and <1%^42]^ and IBD. None of the loci identified in the two studies overlapped or were replicated in other studies. Data are summarised in Table 1 and Figure 3A and B.

**Table 1.** CNVs identified in IBD patients from Decipher entries, case reports, and unselected CNV analyses

Chromosome	Location	Size	Genes	Reference
A. Deletions				
2	2q33.2	606 kb	*CTLA4*, *ICOS*	Tran *et al.*^36^
3	3p26.3q25.3	9.62 Mb	62	Decipher 349797
5	5q35.3	185.2 kb	13	Decipher 308104
6	6p21.31	5.7 kb	*IP6K3*	Frenkel *et al*.^41^
	6p25.3	108.4–113.6 kb	*DUSP22*	Frenkel *et al*.^41^
	6q23.3	119 kb	*TNFAIP3*	Taniguchi *et al*.^37^
7	7p22.1	100.4 kb	*FAM220A*, *RAC1*	Frenkel *et al*.^41^
	7q31.1	173.63 kb	*IMMP2L*	Decipher 390408
10	10p15.1	374 kb	*IL2RA*, *IL15RA*, *FBXO18*, *ANKRD16*, *RBM17*, *GD12* [partial], *PFKFB3* [partial]	Joosse *et al*.^43^
13	13q32.1	15.8 kb	Upstream of *ABCC4* [*MRP4*] and *CLDN10*	Saadati *et al*.^42^
15	15q11.2q13.1	5.33 Mb	132	Decipher 285124
16	16p13.3	2.09 Mb	124	Decipher 249933
	16p13.3	1.9 Mb	88	Cox *et al*.^20^
X	Xq25	55 kb	*XIAP*	Kelsen *et al.*^38^
B. Duplications				
1	1p36.33	9–87 kb	*ACAP3*	Frenkel *et al*.^41^
2	2q33.3q34	4.34 Mb	70	Decipher 333025
3	3q21.3	23.2–78.4 kb	*PLXNA1*	Frenkel *et al*.^41^
6	6q12	263.15 kb	*EYS*	Decipher 278651
7	7q21.3q22.2	7.84 Mb	196	Decipher 349742
	7p22.1	119 kb	Entirety of *ZNF815*, *OCM* and overlaps with *RNF216*, *RSPH10B*	Saadati *et al.*^42^
8	8q24.3	5.9–11.9 kb	*PLEC*	Frenkel *et al*.^41^
	8q24.3	134 kb	Upstream of *KCNK9* [*TASK3*]	Saadati *et al*.^42^
9	9q34.3	29.6–100 kb	*SAPCD2*	Frenkel *et al*.^41^
14	14q32.33	13.9–44.7 kb	*MTA1*, *CRIP2*, *CRIP1*, *C14orf80*	Frenkel *et al.*^41^
15	15q11.2	6–62.2 kb	*SNORD115-6*, *SNORD115-7*, *SNORD115-8* [RNA genes]	Frenkel *et al*.^41^
16	16q22.1-tel	19.3 Mb	249	Decipher 349797
	16p13.3	6–16.2kb	*UBALD1*	Frenkel *et al*.^41^
17	17q25.3	3.47 Mb	111[*SLC25A10*]	Decipher 308104
	17q25.3	6.6-7.6 kb	*SLC25A10*	Frenkel *et al*.^41^
19	19p13.3	12-13.2 kb	*PSPN*, *GTF2F1*	Frenkel *et al*.^41^
20	20q13.11	269.74 kb	5	Decipher 349742
X	Xq27.3	372.9 kb	7	Decipher 249933
C. Triplications				
15	15q26	7.8 Mb	41	Cox *et al*.^20^
21	21q22.11	2.46 Mb	75	Decipher 293457

IBD, inflammatory bowel disease; CNV, copy number variation.

In addition to these systematic studies, there were published case reports and patients with intestinal inflammation reported in clinical databases such as DECIPHER or Clinvar [Table 1, Figure 3A and B]. One published case report and seven DECIPHER database entries described patients with IBD and deletions and/or duplications of more than 1 Mb [Supplementary Table 3]. Five of these patients had very-early onset IBD.

Among genes previously implicated in Mendelian disorders associated with an increased penetrance of IBD, CNV were identified affecting *TRNT1, CYBA*, *ARPC1B*, and *IL2RA* genes. The deletion 3p26.3q25.3 present in one patient spanned the *TRNT1* gene, which has been associated with intestinal inflammation with autosomal recessive defects.^44,45^ The same patient had a duplication 16q22.1 that spanned the *CYBA* monogenic IBD gene and *PLCG2*. Gain-of-function variants in *PLCG2* have been associated with IBD as part of a broader phenotype of PLCγ2-associated antibody deficiency and immune dysregulation [PLAID] or auto-inflammation and PLCγ2-associated antibody deficiency and immune dysregulation [APLAID] syndromes.^46,47^ Another patient with a 7q21.3q22.2 duplication spanned the monogenic IBD gene *ARPC1B*; however intestinal inflammation was previously associated with an autosomal recessive loss-of-function of this gene.^48^ The duplication of the *IL2RA* locus as a potential cause of very-early-onset IBD was described in a 2-year-old patient [Figure 2C, Supplementary Table 3].^43^ Again, although this involves a different mode of inheritance [gene loss rather than gene dose increase], loss-of-function autosomal recessive variants in *IL2RA* have been associated with chronic intestinal inflammation.^49^

Two DECIPHER entries reported patients with IBD and microdeletions/microduplications that spanned a single gene: duplication of the *EYS* gene and a deletion of the *IMMP2L* gene [involved in mitochondrial protein transport]. Pathogenic *EYS* variants are typically associated with retinitis pigmentosa and defective photoreceptor development and the gene also demonstrates enriched expression in intestinal enteroendocrine cells and granulocytes.^50^

The majority of the CNVs identified in all sources were unique to single patients. There were two loci reported in both the literature and identified among the DECIPHER dataset patients. Deletion 16p13.3 was present in the genome of a 3-year-old DECIPHER patient [249933] with CD. A 36-year-old patient with colitis, described by Cox and Butler [2015], had partial trisomy 15q26 and partial monosomy 16p13.3.^20^ These two 16p13.3 deletions had an overlap of 1.92 Mb. The DECIPHER Patient 308104, diagnosed with UC, had a complement duplication17q25.3, which was also overrepresented in the IBD patients investigated by Frenkel *et al.* [2019].^41^ Both duplications overlapped with the *SLC25A10* gene that encodes a mitochondrial transmembrane transporter.

### 3.8. Replication study *IL2RA*

Only one of the recently described duplications, a variant spanning the *IL2RA* locus and presenting with infantile-onset IBD, was supported by functional evidence.^43^ We have therefore chosen this variant for validation analysis. The previously described variant duplication of the 374kb locus included not only *IL2RA*, *IL15RA, FBXO18*, *ANKRD16*, and *RBM17*, but also two partially overlapping genes [*GD12*, *PFKFB3*]. Functional evidence suggested a gene dose effect of the IL2 receptor.^43^ In the replication study and search, we investigated duplications that encompass the entire *IL2RA* gene, assuming that such CNVs likely result in a higher gene dosage.

We found no record of whole-gene *IL2RA* duplication in the gnomAD database, suggesting that this duplication cannot be observed in reference populations around the globe. As the functional validation of the *IL2RA* duplication and its potential role in IBD provided a relevant and testable hypothesis, we further investigated the impact of *IL2RA* duplication variants in the IBD patient population. We performed a survey among multiple paediatric IBD centres for patients where a clinical diagnosis of *IL2RA* duplication was made and searched genetically characterised IBD cohorts in the UK [Oxford and London], screened an IBD cohort using parental samples as controls [Toronto], and searched the DECIPHER database and the 100 000 Genomes Project for further patients.

**Figure 4.**
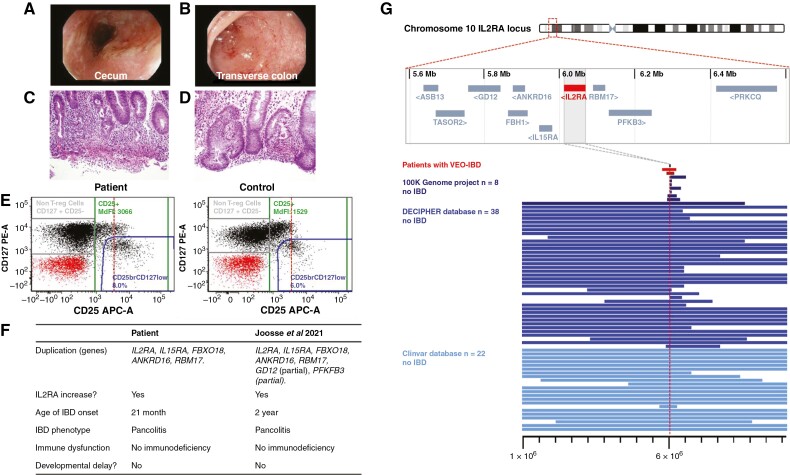
Duplications in the *IL2RA* locus—case report and systematic analysis. Ileocolonoscopy images showing evidence of pancolitis with redness, ulcerations, exudate, and mucosal friability in the caecum [A] and transverse colon [B]. Histological examination revealed [C, D] neutrophilic inflammation including cryptitis and crypt abscesses as well as crypt distortion and basal lymphoplasmacytosis [200x magnification]. [E] Increased CD25 [*IL2RA*] fluorescence intensity in patient-derived CD4 + T cells identified by fluorescence activated cell sorting [FACS]. MdFI is of CD4 + T cells. Dotted red line indicates MdFI of 3066. [F] Comparison of phenotypic features between two patients with very-early-onset inflammatory bowel disease [IBD] and *IL2RA* duplication. [G] Screening for *IL2RA* duplications in four cohorts. Each bar indicates a duplication overlapping the *IL2RA* locus. Bar length indicates the extent of the duplication. Larger duplications are cut off.

Among tertiary referral centre cohorts, the survey identified one patient with an *IL2RA* duplication and infantile-onset IBD where a diagnosis was established by clinical genetics. Genetic panel screening revealed an *IL2RA* duplication that was confirmed by array comparative genomic hybridisation [aCGH]. The identified chr10:5918731-6182347 region of duplication involved *IL15RA* and *IL2RA* as well as *FBXO18*, *ANKRD16*, and *RBM17*. The 3-year-old female patient was diagnosed with IBD at 21 months of age [infantile-onset IBD] when she presented with diarrhoea, haematochezia, and poor appetite. Endoscopy revealed pancolitis with neutrophilic infiltration, crypt abscesses, crypt distortion, and basal lymphoplasmacytosis [Figure 4A–D]. The patient was started on prednisone and sulphasalazine treatment. The patient was corticosteroid-dependent and needed frequent hospitalisations. Subsequent treatments included corticosteroids, antibiotics, and anti-TNF therapy [adalimumab and infliximab], as well as azathioprine, sirolimus, and tacrolimus. We performed immunophenotyping and functional studies to investigate the impact of the *IL2RA* duplication [Figure 4E]. Immunophenotyping suggested normal CD3 + cells [proportion slightly raised 82% with absolute T cell numbers within normal range for patient’s age], an increased proportion and absolute numbers of CD8 + T cells among T cells [35.1% and 1540 Gpt/l, respectively], normal proportion and absolute numbers of regulatory T cells [CD4 + CD25 + CD127 low and *Foxp3* expressing Treg cells], and normal number of CD3-CD19 + B cells.

In line with the *IL2RA* duplication, the median fluorescence intensity [MdFI] of the CD25 signal among CD4 + T cells was about double in the patient compared with control [MdFI 3066 versus 1529; Figure 4E].

The proportion of activated HLA DR + effector T cells was high [22.2%]. The proliferation of CD3 + cells in response to stimulation with anti-CD3 antibody was augmented by addition of exogenous IL2 [50.4% vs 62.5%]. IL-2 induced phosphorylation of STAT5 [pSTAT5] in lymphocytes and CD4 T cells on flow cytometry were comparable.

The patient did not present with additional syndromic features or developmental delay but the disease onset, inflammation, and severity of the inflammatory response is comparable to the previously published patient with a similar duplication^43^ [Figure 4F].

To screen an IBD cohort systematically [Figure 4G] a total of 3158 exomes were analysed, of which 1293 were probands with paediatric onset IBD. We were not able to identify additional IBD patients with a duplication of the *IL2RA* locus, but there was one unaffected mother who had an *IL2RA* duplication [DUP:10:5991252:6557098], which also involved five genes [*IL15RA*, *IL2RA*, *PFKFB3*, *PRKCQ*, and *RBM17*].

A CNV analysis of the DECIPHER database revealed 38 patients with duplications in the *IL2RA* locus, of whom 37 patients had both *IL2RA* and *IL15R* duplications [Figure 4G]. Whereas developmental delay was a common finding in those patients, none of the patients were described as having intestinal inflammation. This suggests that the penetrance of intestinal inflammation due to *IL2RA* duplication is low [<2.6%, 90% confidence interval <0.0001 to 0.12]. The median age of patients from the DECIPHER data at their latest clinical visit was 2 years [mean 5.57 years, *n* = 33], potentially representing a bias if IBD has not developed by this age. In the 100 000 Genomes Project, eight participants were identified to have duplications that included *IL2RA* or part of the *IL2RA* gene [Figure 4G]. None of these participants had colitis listed as a phenotype.

In summary, the duplication of the *IL2RA/IL15RA* locus has been identified in two infantile IBD patients with surprising genetic and phenotypic similarity. The duplication might be functionally relevant but the IBD penetrance is likely to be below 2%. There is currently no indication that the size of the duplication or a combination of genes in this locus has additive or protective effects.

## 4. Discussion

We investigated the association between IBD and a diverse range of rare chromosomal structural and numerical abnormalities. Among the numerical chromosomal abnormalities, Turner syndrome was associated with an increased risk of IBD [approximately 2% penetrance], whereas other numerical chromosomal abnormalities had limited evidence for increased susceptibility. Our analysis suggests that patients with Turner syndrome presented with IBD at a significantly younger age than the classical IBD cohort, but typically not at an extreme young age such as very-early-onset IBD. Turner syndrome is a condition in females that results from the complete or partial loss of one X chromosome, altering the gene dosage of up to 15% of genes on the X chromosome that escape inactivation.^51^ Complete loss of one X chromosome or partial deletion has pervasive effects on the genome, epigenome, and transcriptome of female individuals,^52^ all resulting in changed gene dosages. Individuals with Turner syndrome have a distinguishable RNA expression profile, as well as a distinct autosomal DNA and X-chromosomal methylation profiles compared with karyotypical 46,XY males and 46,XX females.^53^ Theoretically, the increased occurrence of IBD in individuals with Turner syndrome could be attributed to the loss of function of monogenic IBD genes on the X chromosome. A possible candidate could be the *CD40LG* that was identified by Trolle *et al*.^53^ as differentially expressed in Turner syndrome patients. However, the distinctly lower penetrance of IBD in Turner syndrome compared with X-linked monogenic IBD defects in genes like *CYBB*, *FOXP3*, and *WAS* suggests a more complex genetic interplay.^6,54–56^ As the loss of one X chromosome or its parts has broad effects on gene expression across the whole genome, development of IBD and other autoimmune disease in patients with Turner syndrome could be due to cumulative gene dosage effects on multiple loci. Alternatively, the findings of co-occurring Turner syndrome and IBD in women with mosaic karyotypes could be biased: a large scale Biobank study showed that patients with mosaic Turner karyotypes have reduced penetrance of comorbidities common in patients with Turner syndrome.^57^ Women with mosaic 45,X/46,XX karyotype were only slightly shorter than average, had a normal birth rate and reproductive lifespan, and had no cardiovascular abnormalities. In our data, this karyotype was rare [*n* = 2 case reports] and other karyotypes were predominant, suggesting that IBD has increased penetrance in non-mosaic Turner syndrome karyotypes.

A well-known contributor of chromosomal abnormalities is the hemizygotic, haploinsufficient or compound heterozygous deletion of established monogenic IBD genes such as *CTLA4*, *ICOS*, *TNFAIP3*, and *XIAP*. In these cases, loss of additional genes might contribute to additional functional defects. Tran *et al.*^36^ discuss a patient with a deletion that encompasses *CTLA4* as well as *ICOS*, presenting with very-early-onset [VEO]-IBD unresponsive to conventional therapy.^36^ Biallelic mutations in the *ICOS* gene cause a deficiency that is characterised by nearly absent class-switched memory B cells; this leads to recurrent infections and autoimmune pathologies including IBD.^58^ The development of VEO-IBD in this patient could be evidence of the causative *CTLA4* deletion, with additional effects of the compound *ICOS* deletion resulting in a more severe IBD phenotype.

The only duplication with functional assessment of the gene dosage consequences was the duplication of the *IL2RA* locus found in a patient with infantile IBD.^43^ We identified another patient with a nearly identical duplication. However, our systematic search for additional patients with *IL2RA* CNV suggests that the duplication of the *IL2RA* locus is associated with expressivity of infantile IBD that is <2%. Absent *IL2RA* signalling causes an immune dysregulation, polyendocrinopathy, enteropathy, and X-linked [IPEX]-like condition due to impaired persistence and function of regulatory T cells.^49^ Joosse *et al*.^43^ showed that the duplication increases IL2 responsiveness in activated CD4 + T cells, which they postulated could then overstimulate these cells in the antigen-rich environment of the colon and induce inflammation. Our functional data support an effect on effector T cells since those show stronger IL2RA expression. However, the DECIPHER data illustrate that duplication of the *IL2RA* locus is consistently associated with developmental problems, but not with IBD or other immune-mediated disorders. Our screening of multiple cohorts makes a selection bias [patients with a syndromal phenotype might be underrepresented in IBD cohorts] or reporting bias [IBD might be underreported in databases such as DECIPHER] less likely. In light of the very variable duplication size flanking the *IL2RA* locus from both sides, it is unlikely that microduplications including the *IL2RA* and *IL15RA* locus are compensated by a dose effect of protective genes. We cannot exclude that additional genetic variants [such as somatic variants] contribute to the IBD risk in patients with *IL2RA* locus duplication.

In summary, we found evidence that some numerical chromosomal aberrations like the Turner syndrome might contribute to an IBD phenotype. The role of other numerical abnormalities and structural abnormalities is less clear. Copy number analysis is essential in patients where a monogenic IBD cause needs to be excluded. Our study highlights the complexities involved with analysing gene copy number data: a large systematic analysis of copy number variation in a large set of patients is required to gain better insight into how chromosomal aberrations contribute to IBD.

## Supplementary Material

jjac103_suppl_Supplementary_Data

## Data Availability

All data described are available in deposited databases [such as Decipher or GEL] or are available on request.
