# Supplementary material for: Chromosomal Numerical Aberrations and Rare Copy Number Variation in Patients with Inflammatory Bowel Disease
Source: J Crohns Colitis. 2022 Jul 30;17(1):49–60. doi: 10.1093/ecco-jcc/jjac103 (PMC9880952; doi:10.1093/ecco-jcc/jjac103)
Supplement: jjac103_suppl_Supplementary_Data [file jjac103_suppl_supplementary_data.docx]

**Supplementary Figures and Tables.**

**Supplementary Table 1.** Patients with full trisomy or monosomy conditions and IBD.

| Chromosomal abnormality | Number of cases (patients) | IBD phenotype (age of onset IBD, gender) | Publication |
| --- | --- | --- | --- |
| Trisomy 21 | N=1  (out of 102) | Ulcerative colitis* | Baccichetti *et al*. ^1^ |
|  | Case report | 16-year-old male, CD  Comorbid primary sclerosing cholangitis (PSC) | Vajro *et al*. ^2^ |
|  | N=1 (out of 2) | 39-year-old male, CD  Comorbid PSC | Kaushik *et al*. ^3^ |
|  | Case report | 8-year-old female, CD | Persic *et al*. ^4^ |
|  | Case report | 6-year-old female, CD | Yamamoto *et al*. ^5^ |
|  | Cross-sectional study; N=6 (out of 1453) | CD (n=3) and UC (n=3).* | Goldacre *et al.* ^6^ |
|  | N=1 (out of 57) | IBDU* | Wallace ^7^ |
|  | N=4 (out of 4) | Female patients with CD, mean age of diagnosis – 13*. NOD2/CARD15 negative. | Morris *et al*. ^8^ |
|  | N=3 (out of 3) | 24-year female with UC (C: abdominal sepsis, toxic megacolon); 27-year male with CD, 34 year male with IBDU* | Souto-Rodriguez *et al*. ^9^ |
|  | Case report | 5-year-old female with CD and secondary lung nodules | Thaver *et al*. ^10^ |
|  | Case report | 10-year-old male with CD. | Gatti *et al.* ^11^ |
| Trisomy 9 (mosaic) | Case report | 2-year-old male with CD | Wooldridge *et al*. ^12^ |
| Trisomy 16 (mosaic) | Case report | 10-year-old female with UC | Abell *et al*. ^13^ |
| Trisomy 18 | Case report | 3-year-old | This manuscript |
| Klinefelter syndrome | N=5 (out of 2208) | Patients with UC | Seminog *et al.* ^14^ |
| Monosomy X – Turner syndrome | Case reports (N=5) | 1. 45,X/46,XX 13-year-old female with UC requiring surgery 2. 46,X,i(Xq) (predominant) 9-year-old female with CD 3. 45,X 14-year-old female with CD 4. 45,X/46,X,i(Xq) 17-year-old female with CD 5. 45,X/46,X,i(Xq) 41-year-old female with UC requiring surgery. | Arulanantham *et al*. ^15^ |
|  | Case reports (N=2) | 1. 46,X,i(Xq) 18-year-old female with CD and comorbid arthritis 2. 45X/46XX 15-year-old female with CD (C: fistula requiring surgery) | Kohler *et al*. ^16^ |
|  | Case report | 46,X,i(Xq) 31-year-old female with UC and comorbid Hashimoto’s thyroiditis | Nishimura *et al*. ^17^ |
|  | Case report | 45,X/47,XY,+13 6-year-old (onset/diagnosis) female with CD | Knudtzon *et al*. ^18^ |
|  | Case report | 45,X/46,X,i(Xq) 26-year-old female with CD (C: intestinal fistulas requiring surgery) | Knudtzon *et al*. ^19^ |
|  | Case report | 45,X/46,X,i(Xq) 22-year-old female with CD | Bamba *et al*. ^20^ |
|  | Case report | 45,X 6-year-old female diagnosed with IBDU. Comorbid coeliac disease and PSC | Lacaille *et al*. ^21^ |
|  | Case report | 46,X,i(Xq) 13-year-old female with CD. | Hayward *et al.* ^22^ |
|  | Case report | 45,X/47,XXX 16-year-old female diagnosed with CD | Tajima *et al*. ^23^ |
|  | Case report | 45,X 35-year-old female with IBDU*. Comorbid von Willebrand disease and primary biliary cholangitis | Sokol *et al*. ^24^ |
|  | Case report | 45,X 29-year-old female with CD. Comorbid coeliac disease | Durusu *et al*. ^25^ |
|  | Case report | 45,X/47,XXX 15-year-old female with CD | Ohkawara *et al*. ^26^ |
|  | Case report | 45,X 4-year-old female with UC | Takaya *et al*. ^27^ |
|  | Case report | 46,X,i(Xq) 13-year-old female with UC | Hyodo *et al*. ^28^ |
|  | N=11 (out of 798) | Female patients with CD (n=3) and UC (n=8)* | Jorgensen *et al*. ^29^ |
|  | Case report | 45,X 24-year-old female with CD. Comorbidity Hashimoto’s thyroiditis. | Triantafillidis *et al*. ^30^ |
|  | N=9 (out of 224) | Female patients with CD (n=5) and UC (n=4)* | Bakalov *et al*. ^31^ |
|  | N=45 (out of 2459) | Female patients with CD (n=27) and UC (n=18)* | Goldacre *et al*. ^32^ |
|  | Case report | 45,X/46,XY female with CD* | Tas *et al*. ^33^ |
|  | N=7 (out of 385) | Female patients with CD (n=3) and UC (n=4)* | Hanew *et al*. ^34^ |
|  | Case report | 32-year-old female with CD. | Keating *et al.* ^35^ |
|  | Case report | 45,X/46,XX 3-year-old female with UC. | Gatti *et al*. ^11^ |

**Supplementary Table 2.** Examples of microdeletions and microduplications.

| **Chromosomal abnormality** | **Number of cases (patients)** | **IBD phenotype (age of onset IBD, gender)** | **Publication/DECIPHER patient number** |
| --- | --- | --- | --- |
| *IL2RA* duplication (374kb) | Case report | 2-year-old female with IBD, therapy resistant, required colectomy | Joosse et al. ^36^ |
| *CTLA-4 & ICOS* deletion (606kb) | Case report | 6-year-old female with IBD, therapy resistant | Tran et al. ^37^ |
| *TNFAIP3* deletion (119kb) | Case report | 6-months-old female with IBD, perianal fistulae | Taniguchi et al. ^38^ |
| *XIAP* deletion (55kb) | Case report | 3-weeks-old male with IBD, therapy resistant, remained severe after colectomy | Kelsen et al. ^39^ |
| Dup6q12 (EYS) | Case report | Female with UC* | DECIPHER patient 278651 |
| Del7q31.1 (IMMP2L) | Case report | 11-year-old female with UC | 390408 |

*Unspecified age of IBD onset and/or gender

**Supplementary Table 3.** Examples of partial trisomy or monosomy conditions.

| **Chromosomal abnormality** | **Number of cases (patients)** | **IBD phenotype (age of onset IBD, gender)** | **Publication/DECIPHER patient number** |
| --- | --- | --- | --- |
| Partial Trisomy 15q26 and Partial Monosomy 16p13.3 | Case report | 36-year-old male with colitis | Cox et al. ^40^ |
| Del15q11.2q13.1 (5.33Mb) | Case report | 2-year-old male with colitis | DECIPHER patient 285124 |
| Triplication 21q22.11  (2.46Mb) | Case report | 14-year-old male with enterocolitis  Comorbid immunodeficiency & psoriasiform dermatitis | 293457 |
| Duplication17q25.3 (3.47Mb)  Del5q35.3 (185.2kb) | Case report | 1-year-old male with UC  Comorbid asthma | 308104 |
| Duplication20q13.11 (269.74kb)  Duplication7q21.3q22.2  (7.84Mb) | Case report | 7-year-old with enterocolitis* | 349742 |
| Del3p26.3p25.3 (9.62Mb)  Duplication16q22.1 (terminal) (19.3Mb) | Case report | Infant (<1-year-old) with enterocolitis* | 349797 |
| Del16p13.3 (2.09Mb)  DuplicationXq27.3 (372.9kb) | Case report | 3-year-old male with CD | 249933 |
| Dup2q33.3q34 (4.34Mb) | Case report | Infant (<1-year-old) with CD* | 333025 |

**Supplementary Figure 1.** Search strategies used for all conditions.

| \| **Pubmed** \| \| --- \| \| **Turner syndrome** \| \| #1 Turner syndrome [MH] \| \| #2 inflammatory bowel disease [MH] \| \| #3 #1 AND #2 \| \| **Down syndrome** \| \| #4 Down syndrome [MH] \| \| #5 #4 AND #2 \| \| **Partial conditions** \| \| #7 “partial trisomy” OR “partial monosomy” \| \| #8 #7 AND #2 \| \| **CNVs** \| \| #9 copy number variation [MH] \| \| #10 #9 AND #2 \| | \| **Embase** \| \| --- \| \| **Turner syndrome** \| \| #1 “Turner syndrome”.tw. \| \| #2 “Monosomy X”.tw. \| \| #3 “Ullrich-Turner Syndrome”.tw. \| \| #4 inflammatory bowel disease/ or Crohn disease/ or ulcerative colitis/ \| \| #5 (1 or 2 or 3) and 4 \| \| **Down syndrome** \| \| #6 Down syndrome/ \| \| #7 4 and 6 \| \| **Partial conditions** \| \| #8 “partial trisomy/ or “partial monosomy/ \| \| #9 4 and 8 \| \| **CNVs** \| \| #10 copy number variation/ \| \| #11 neoplasm/ or malignant neoplasm/ or cancer/ \| \| #12 (4 and 10) not 11 \| |
| --- | --- | --- | --- | --- | --- | --- | --- | --- | --- | --- | --- | --- | --- | --- | --- | --- | --- | --- | --- | --- | --- | --- | --- | --- | --- | --- | --- | --- | --- | --- | --- | --- |

**Supplementary Information: Trisomy 18 and intestinal inflammation**

The female infant was born at 36 weeks gestation with a birth weight of 1840 g (3rd centile). She was found to have a double-chambered right ventricle and a persistent foramen ovale. Trisomy 18 was diagnosed postnatally.

By 2 years of age neurodevelopmental delay was severe, and she had seizures, which were managed with levetiracetam. Cardiovascular status was stable without medications . Linear and ponderal growth were impacted (weight 8.3 kg, < 0.4th centile; length 84 cm, 3rd centile; head circumference 44.6 cm, 1st centile), and she was exclusively enterally fed with an amino acid-based formula via gastrostomy

Following a urinary tract infection managed with oral antibiotics (Cefpodoxim) she developed bloody diarrhoea. At the age of 2 ½ years she was admitted for diagnostic work up to investigate for possible intestinal inflammation. At this time, serum CRP was not raised but stool calprotectin was elevated (960 μg/g), and she was anaemic (haemoglobin 9.0 g/dl). Extensive microbiology investigations did not detect an infection (this included stool cultures, serology for Yersinia and cytomegalovirus and stool cultures for pathogens including Clostridium difficile). Sigmoidoscopy revealed multiple erosions and haemorrhagic lesions. Histology identified discontinuous inflammation dominated by lymphocytes and plasma cells, some eosinophils and neutrophils, but no granulomas. Intestinal crypts were slightly deformed. The diagnostic label fits with Inflammatory Bowel Disease unclassified (IBDU). Treatment with sulfasalazine and prednisolone resolved the diarrhoea and rectal bleeding. A relapse was treated successfully by prednisolone.

**Supplementary References**

1. Baccichetti C, Lenzini E, Pegoraro R. Down syndrome in the belluno district (veneto region, northeast italy): Age distribution and morbidity. *Am J Med Genet Suppl* 1990;**7**:84-6.

2. Vajro P, Cucchiara S, Vegnente A*, et al.* Primary sclerosing cholangitis preceding crohn's disease in a child with down's syndrome. *Dig Dis Sci* 1998;**43**:166-9.

3. Kaushik SP, Kaye G, Clarke AC. Autoimmune hepatobiliary disease in trisomy 21. *J Clin Gastroenterol* 2000;**30**:330-2.

4. Persic M, Dessardo S, Subat-Dezulović M, Ahel V, Rozmanić V. Down syndrome and crohn's disease: An extremely rare association. *Pediatr Int* 2001;**43**:519-21.

5. Yamamoto M, Abo W, Hori T*, et al.* Crohn's disease in a child with down syndrome. *Pediatr Int* 2002;**44**:537-9.

6. Goldacre MJ, Wotton CJ, Seagroatt V, Yeates D. Cancers and immune related diseases associated with down’s syndrome: A record linkage study. *Archives of Disease in Childhood* 2004;**89**:1014.

7. Wallace RA. Clinical audit of gastrointestinal conditions occurring among adults with down syndrome attending a specialist clinic. *J Intellect Dev Disabil* 2007;**32**:45-50.

8. Morris D, Ashai-Khan F. Nod2/card15 negative crohn's disease in a cohort of down syndrome patients. *American Journal of Gastroenterology* 2011;**106**:S402.

9. Souto-Rodríguez R, Barreiro-de-Acosta M, Domínguez-Muñoz JE. Down's syndrome and inflammatory bowel disease: Is there a real link? *Rev Esp Enferm Dig* 2014;**106**:220-2.

10. Thaver D, Beg M. Pulmonary crohn's disease in down syndrome: A link or linkage problem. *Case Rep Gastroenterol* 2016;**10**:206-11.

11. Gatti S, Gelzoni G, Catassi GN, Catassi C. The clinical spectrum of inflammatory bowel disease associated with specific genetic syndromes: Two novel pediatric cases and a systematic review. *Frontiers in Pediatrics* 2021;**9**.

12. Wooldridge J, Zunich J. Trisomy 9 syndrome: Report of a case with crohn disease and review of the literature. *Am J Med Genet* 1995;**56**:258-64.

13. Abell R, Difalco J, Morganstern J. Ulcerative colitis in a child with partial trisomy 16. *Journal of Crohn's and Colitis* 2013;**7**:e403-e.

14. Seminog OO, Seminog AB, Yeates D, Goldacre MJ. Associations between klinefelter’s syndrome and autoimmune diseases: English national record linkage studies. *Autoimmunity* 2015;**48**:125-8.

15. Arulanantham K, Kramer MS, Gryboski JD. The association of inflammatory bowel disease and x chromosomal abnormality. *Pediatrics* 1980;**66**:63-7.

16. Kohler JA, Grant DB. Crohn's disease in turner's syndrome. *Br Med J (Clin Res Ed)* 1981;**282**:950.

17. Nishimura H, Kino M, Kubo S, Kawamura K. Hashimoto's thyroiditis and ulcerative colitis in a patient with turner's syndrome. *Jama* 1985;**254**:357.

18. Knudtzon J, Ledaal P, Middelthon-Moe M, Aarskog D. 45,x/47,xy,+13 mosaicism and crohn's disease. *Acta Paediatr Scand* 1988;**77**:922-4.

19. Knudtzon J, Svane S. Turner's syndrome associated with chronic inflammatory bowel disease. A case report and review of the literature. *Acta Med Scand* 1988;**223**:375-8.

20. Bamba T, Hashiguchi M, Doi H*, et al.* A case of turner's syndrome with crohn's disease. *Digestive Endoscopy* 1993;**5**:263-8.

21. Lacaille F, Canioni D, Bernard O*, et al.* Celiac disease, inflammatory colitis, and primary sclerosing cholangitis in a girl with turner's syndrome. *Journal of Pediatric Gastroenterology and Nutrition* 1995;**21**.

22. Hayward PAR, Satsangi J, Jewell DP. Inflammatory bowel disease and the x chromosome. *QJM: An International Journal of Medicine* 1996;**89**:713-8.

23. Tajima T, Oishi M, Nakae J*, et al.* Turner syndrome with crohn disease. *Clinical Pediatric Endocrinology* 2001;**10**:121-4.

24. Sokol L, Stueben ET, Jaikishen JP, Lamarche MB. Turner syndrome associated with acquired von willebrand disease, primary biliary cirrhosis, and inflammatory bowel disease. *Am J Hematol* 2002;**70**:257-9.

25. Durusu M, Gürlek A, Simşek H, Balaban Y, Tatar G. Coincidence or causality: Celiac and crohn diseases in a case of turner syndrome. *Am J Med Sci* 2005;**329**:214-6.

26. Ohkawara T, Takeda H, Miyashita K*, et al.* Crohn's disease in turner's syndrome with x-chromosomal mosaicism of 45 xo and 47 xxx. *J Gastroenterol* 2005;**40**:914-6.

27. Takaya J, Teraguchi M, Ikemoto Y*, et al.* Turner syndrome associated with ulcerative colitis. *Clinical Pediatric Endocrinology* 2006;**15**:97-100.

28. Hyodo H, Tomita Y, Hirai K*, et al.* Turner syndrome with ulcerative colitis. *Clinical Pediatric Endocrinology* 2009;**18**:101-5.

29. Jørgensen KT, Rostgaard K, Bache I*, et al.* Autoimmune diseases in women with turner's syndrome. *Arthritis & Rheumatism* 2010;**62**:658-66.

30. Triantafillidis JK, Nadia el F, Fostira F*, et al.* Turner's syndrome, autoimmune thyroiditis, and crohn's disease in the same patient: A combination emphasizing the role of x-chromosome in inflammatory bowel disease patients. *Inflamm Bowel Dis* 2010;**16**:1088-9.

31. Bakalov VK, Gutin L, Cheng CM*, et al.* Autoimmune disorders in women with turner syndrome and women with karyotypically normal primary ovarian insufficiency. *J Autoimmun* 2012;**38**:315-21.

32. Goldacre MJ, Seminog OO. Turner syndrome and autoimmune diseases: Record-linkage study. *Archives of Disease in Childhood* 2014;**99**:71-3.

33. Tas E, Yatsenko SA, Popovic J. 46, xy female with turner syndrome, gonadoblastoma, crohn's disease and low level mosaicism for monosomy x. *Endocrine Reviews Conference: 97th Annual Meeting and Expo of the Endocrine Society, ENDO* 2015;**36**.

34. Hanew K, Tanaka T, Horikawa R, Hasegawa T, Yokoya S. Prevalence of diverse complications and its association with karyotypes in japanese adult women with turner syndrome-a questionnaire survey by the foundation for growth science. *Endocr J* 2018;**65**:509-19.

35. Keating E, Kelleher TB, Lahiff C. De novo anti-tnf-α-induced congestive heart failure in a patient with turner syndrome and crohn’s disease. *Inflammatory Bowel Diseases* 2020;**26**:e161-e2.

36. Joosse ME, Charbit-Henrion F, Boisgard R*, et al.* Duplication of the il2ra locus causes excessive il-2 signaling and may predispose to very early onset colitis. *Mucosal Immunology* 2021.

37. Tran NN, Setty M, Cham E, Chan AY, Ali S. Ctla-4 haploinsufficiency presenting as extensive enteropathy in a patient with very early onset inflammatory bowel disease. *JPGN Reports* 2021;**2**:e099.

38. Taniguchi K, Inoue M, Arai K*, et al.* Novel tnfaip3 microdeletion in a girl with infantile-onset inflammatory bowel disease complicated by a severe perianal lesion. *Human Genome Variation* 2021;**8**:1.

39. Kelsen JR, Dawany N, Martinez A*, et al.* A de novo whole gene deletion of xiap detected by exome sequencing analysis in very early onset inflammatory bowel disease: A case report. *BMC Gastroenterol* 2015;**15**:160.

40. Cox DM, Butler MG. Distal partial trisomy 15q26 and partial monosomy 16p13.3 in a 36-year-old male with clinical features of both chromosomal abnormalities. *Cytogenetic and Genome Research* 2015;**145**:29-34.
